# Multiple social disadvantage does it have an effect on amenable mortality: a brief report

**DOI:** 10.1186/s12939-014-0067-5

**Published:** 2014-08-01

**Authors:** Kristiina Manderbacka, Martti Arffman, Reijo Sund, Sakari Karvonen

**Affiliations:** 1 National Institute for Health and Welfare, Service System Research Unit, Helsinki, FI-00271, Finland; 2 Department of Social and Health Policy and Economics, Helsinki, FI-00271, Finland

**Keywords:** Social disadvantage, Social inequality, Amenable mortality

## Abstract

**Introduction:**

Most studies on inequalities in health and health-care focus on single indicators of social position, e.g. income or education. Recent research has suggested that multiple social circumstances need to be analysed simultaneously to disentangle their influence on health. In past decades mortality amenable to health-care, i.e. premature mortality that should not occur given timely and effective health-care, has increasingly been used to study the effect of health-care on health outcomes. This study elaborates the effect of social and regional deprivation and unemployment on the association between income and mortality amenable to health-care in Finland.

**Methods:**

Individual-level data for deaths were gathered by disease category between 1992 and 2008 for the resident Finnish population aged 25 to 59 years. Differences in amenable mortality and changes over time were assessed using individual-level linked register data. We used gender- and age-standardised rates and Poisson regression models to examine the simultaneous effect of these indicators on amenable mortality.

**Results:**

Altogether 22,663 persons aged 25–59 years died from causes amenable to health-care during the study period. An inverse pattern was found in amenable mortality for income. The mortality rate in the lowest income quintile was 98 (93–104) per 100,000 in the period 1991–1996 while in the highest group the figure was 40 (38–42) for the same period. Whereas the level of amenable mortality decreased, mortality differences between income groups steepened and amenable mortality increased in the lowest income group towards the end of the study period. Those in poor labour market position or living alone had significantly larger income differences in amenable mortality. Risk of regional deprivation was not associated with amenable mortality.

**Conclusions:**

In order to prevent and treat at an early phase conditions that otherwise may lead to premature and unnecessary deaths more attention should be focused on groups with increased social and economic deprivation risk in municipal health centres with the aim at improving access to primary care. Our results also call for joint action by both health-care and social services, since health services alone cannot deal with the risks posed by accumulating social disadvantage.

## Introduction

Studies on inequalities in health and access to health-care mainly focus on single indicators of social position, such as income, occupational position or level of education. Recently, however, the persistence of inequalities and their cross-cultural invariance have led researchers to state that in order to understand the root causes, we need to analyse in more detail the intersections of social inequalities and to analyse multiple social circumstances simultaneously to disentangle their influence on health and access to health-care [1]–[3]. There is also an abundance of evidence showing how disadvantage accumulates due to lack of economic resources and regionally though it is still relatively poorly understood how the different indicators of social diversity interact [4],[5]. There is evidence from earlier studies both in Finland [6],[7], and elsewhere [8] that disadvantage from economic and social deprivation and being unemployed accumulates and also on accumulation of poverty and unemployment in sparsely populated rural areas both internationally [9] and in Finland [10].

In addition to material or social and psychological circumstances, health-care can influence health outcomes. The structure of the Finnish health-care system, in general, supports equal access to health-care. The system is mainly financed by tax revenues, while user-fees are, in general, low [11]. There are, however, some parts of the system that do not support equal access. Whereas ambulatory services are primarily provided by the public sector (e.g. 38% of visits to the doctor in 2009) and financed through taxation there have been difficulties in access. Private ambulatory services (23% of visits) are available especially in cities and larger municipalities, but patients’ co-payments are high. Occupational health-care (24% of visits) provides easy access to ambulatory care for employees free of charge. The hospital districts – all municipalities in Finland belong to a local hospital district – organise and provide specialist medical services for the residents and are managed and funded by the municipalities. While general practitioners act as gate-keepers for public specialist services in the public sector and in occupational health-care, no gate-keeping is exercised in private services.

In the last decades, mortality amenable to health-care, i.e. premature mortality that should not occur given timely and effective health-care, has increasingly been used to study the effect of health-care on health outcomes [12]–[14]. Although studies on amenable mortality have used slightly different lists of amenable conditions and different age bands and originate in different countries and time periods, the main results have been largely similar. The studies have reported differences in amenable mortality between genders [15]–[17], ethnic groups [18]–[20], immigrants compared to native population [21],[22], marital status groups [15], between socioeconomic groups [15],[16],[19],[23],[24], by employment status [15] and regionally [15],[23],[25]. A recent study from Finland has reported large and increasing income group differences in amenable mortality from the 1990s to 2008 [26]. As the majority of earlier studies on amenable mortality have identified the associations by using single indicators of social position, less is known of their intersections.

The aim of the present study is to elaborate income inequalities in amenable mortality in Finland among the working age population aged 25 to 59 years. In addition to the economic dimension measured by income, we analyse three other dimensions of socioeconomic position that reflect an increased risk for social disadvantage, namely social deprivation, regional deprivation and unemployment. Our main focus is to analyse the effect of these dimensions on the association between income and mortality amenable to health-care. We also analyse changes in time of the associations between these dimensions of social position and amenable mortality during the period 1992 to 2008.

## Methods

### The data

Differences in mortality amenable to health-care and changes over time from 1992 to 2008 were assessed using individual-level linked register data. Data were extracted from the Causes of Death Statistics of Statistics Finland for the population aged 25 to 59 years considering deaths by disease category, including external causes between 1992 and 2008. While the official retirement age in Finland is 63 years, the expected effective retirement age was 59.4 years in 2008 [27]. In order to be able to analyse the effect of unemployment on mortality, we selected the resident Finnish population aged 25 to 59 years in each year as the annual population at risk to minimise the effect of retirement on the results. The study period covered the transition from ICD-9 to ICD-10 in 1996. We classified deaths amenable to health-care based on the Nolte and McKee [12] list, which was supplemented with infections that are preventable by hygiene measures or vaccination, asthma and COPD, which are to an extent both preventable and effectively treatable especially in younger age groups, and benign tumours and malignant neoplasm of the bladder, which, it has been suggested, are treatable in the main [13] (see Additional file 1).

The long-term institutionalised population (1.5% of the study population) as well as retired persons (6.2% of the study population) were excluded from the analyses, since not all their socioeconomic variables could be obtained from the registers used. Indicators for income, living arrangements and region of residence were measured yearly on the basis of information from the year preceding each follow-up year. We used income as an indicator of the economic dimension. Income quintiles were calculated for family net income and adjusted for family size using the OECD equivalence scale [28]. Social deprivation risk was measured by examining those living alone vs. others. For our indicator of regional deprivation, region of residence was classified to cities, rural areas near cities, core rural areas and sparsely populated rural areas, the latter of which was considered to increase deprivation risk. Region of residence was also classified to five University hospital districts. For our indicator of unemployment, employment status was classified as working, short-term unemployed (less than 10 months/year) and long-term unemployed (10–12 months/year).

### Statistical methods

Gender and age-standardised rates were calculated for mortality amenable to health-care in each of the indicators that increase the risk of disadvantage. A direct method of standardisation with five-year age bands was used. Poisson regression models were used to examine the simultaneous effect of these indicators on amenable mortality. In order to handle multicollinearity and interactions found in preliminary analyses between the indicators, we decided to stratify the Poisson models by living arrangements. Of the remaining variables the condition index between income and employment status remained expectedly quite high (17.3). As interactions with time were found, we used two approaches to deal with time in stratified models: 1) models where the time period was adjusted as well as 2) separate models for each of the time periods (1992–96, 1997–002 and 2003–2008) to assess changes in amenable mortality. In all of the models, we included variables for age, gender, income quintile, employment status and interaction of the latter two. Results of the models are presented as contrasts in the combinations by income quintile and employment status compared to those in the highest income group and with no unemployment months. The statistical significance of change over time was tested by including interaction terms between study period, income and employment status in the models. We also tested whether the differences between the highest and lowest income quintiles were similar for those living alone or living with someone in each of the employment status groups separately by using appropriate contrasts to define corresponding tests in the Poisson regression model.

The study protocol was approved by the Research Ethics Committee of the National Institute for Health and Welfare.

## Results

Altogether 24,061 persons aged 25–59 years died in Finland for causes amenable to health-care during the study period. Overall, the amenable mortality rate was 60 (58–61) per 100,000 person years in 1992–96 and 45 (44–46) in 2003–2008. While amenable mortality decreased in this age group during the study period, all the examined indicators of risk of economic and social disadvantage were associated with amenable mortality (Table 1). In 1992–96, the mortality rate in the lowest income quintile was 98 (93–104) per 100,000 while in the highest group the figure was 40 (38–42). A stepwise pattern of increasing amenable mortality by decreasing income could be seen from the highest to the lowest income quintile. Whereas amenable mortality decreased rapidly in higher income groups, it did not decline in the lowest income group during the study period. In 2003–2008 the amenable mortality rate in the lowest income group was four times that of the highest income group. Unemployment and especially long-term unemployment was also associated with increased mortality rates. As with those with a low level of income, amenable mortality did not decline among those in long-term unemployment. Amenable mortality declined both among those living alone and among those living with others, but the mortality rates were considerably higher among those living alone throughout the study period. In contrast to economic and social disadvantage, regional deprivation was not associated with amenable mortality.

**Table 1:** Mortality amenable to health-care among 25–59 -year old Finnish population in 1992–2008

		**1992 − 1996**		**1997 − 2002**		**2003 − 2008**	
		**Deaths**	**Stand rate**	**Deaths**	**Stand rate**	**Deaths**	**Stand rate**
			**(95% CI)**		**(95% CI)**		**(95% CI)**
Income	Lowest	1427	98 (93, 104)	2068	106 (101, 110)	2218	102 (98, 107)
	2	1269	84 (79, 89)	1571	83 (79, 87)	1323	66 (62, 69)
	3	1240	61 (57, 64)	1398	55 (52, 57)	1255	46 (43, 48)
	4	1452	52 (50, 55)	1553	42 (40, 44)	1355	33 (31, 35)
	Highest	1541	40 (38, 42)	1594	31 (29, 32)	1399	25 (24, 27)
Living arrangements	Living alone	1946	99 (95, 104)	2394	93 (90, 97)	2569	80 (76, 83)
	Living with other/s	6381	53 (52, 55)	5790	45 (44, 46)	4981	37 (36, 38)
Employment status	Working	2999	32 (30, 33)	2735	26 (25, 27)	2630	22 (21, 23)
	Unemployed <10 months	633	44 (40, 47)	583	37 (34, 41)	518	36 (33, 39)
	Unemployed 10–12 months	500	63 (57, 68)	747	71 (66, 77)	516	72 (65, 79)
Municipality type	Cities	4843	60 (58, 62)	4939	54 (53, 56)	4426	45 (44, 47)
	Rural areas near cities	1166	55 (52, 59)	1149	48 (45, 51)	1090	41 (38, 43)
	Core rural areas	1455	62 (59, 65)	1272	51 (48, 53)	1245	46 (44, 49)
	Sparsely populated rural areas	863	63 (58, 67)	824	59 (55, 63)	789	54 (51, 58)
University hospital district	Åland Islands	39	60 (41, 79)	35	44 (30, 59)	25	29 (17, 40)
	Helsinki (South)	2685	60 (57, 62)	2780	54 (52, 56)	2549	46 (44, 48)
	Kuopio (East)	1510	63 (60, 66)	1471	57 (54, 60)	1352	49 (47, 52)
	Oulu (North)	1101	58 (55, 62)	1119	54 (51, 57)	1013	45 (42, 48)
	Tampere (West)	1922	60 (57, 63)	1761	50 (48, 52)	1689	44 (42, 47)
	Turku (South-West)	1070	57 (54, 61)	1018	50 (47, 53)	922	42 (40, 45)
Total		8327	60 (58, 61)	8184	53 (52, 54)	7550	45 (44, 46)

Gender and age-standardised rates per 100,000.

When modelling the effects of the three indicators of economic and social disadvantage while controlling for gender and age, all three were statistically significantly associated with amenable mortality, though several interactions were detected between them, as well as between them and time period. No three-way interactions were found in the analyses. Table 2 presents the risk ratios for income differences in amenable mortality by employment status in the three time periods in models stratified by living arrangements, since an interaction was detected between income group and time (p < .0001). In general, a stepwise pattern was found by income: the lower the income the higher the risk for amenable mortality. Income differences in amenable mortality were steeper towards the end of the study period. An interaction effect was also detected between income and living arrangements (p < .0001). Those with low income had higher amenable mortality risk if living alone. This pattern remained similar throughout the study period. An interaction effect was also found between income and unemployment (p < .0001). Income differences in amenable mortality were rather small among those at work. Unemployment steepened the income group differences: those with a low income had a higher risk of mortality if unemployed and especially if long-term unemployed. This was the case throughout the study period among both those living alone and those living with others.

**Table 2:** The association of income, employment status and living arrangements to mortality amenable to health-care

	**Living in 2+ person household**			**Living alone**		
		**Unemployed**			**Unemployed**	
	**Working**	**<10 months**	**10-12 months**	**Working**	**<10 months**	**10-12 months**
**Income**	**RR 95% CI**	**RR 95% CI**	**RR 95% CI**	**RR 95% CI**	**RR 95% CI**	**RR 95% CI**
1992 − 1996						
Highest	1.00	1.00 (0.73, 1.38)	0.48 (0.20, 1.12)	1.00	1.67 (1.01, 2.77)	0.83 (0.11, 6.42)
4	1.08 (0.95, 1.22)	1.13 (0.86, 1.48)	1.13 (0.75, 1.69)	1.34 (1.06, 1.69)	1.50 (0.94, 2.40)	1.07 (0.33, 3.49)
3	0.99 (0.86, 1.16)	1.09 (0.83, 1.43)	2.00 (1.52, 2.63)	1.24 (0.84, 1.84)	2.09 (1.42, 3.07)	1.10 (0.55, 2.20)
2	1.12 (0.94, 1.33)	1.41 (1.09, 1.84)	2.01 (1.54, 2.63)	2.13 (1.35, 3.36)	1.81 (1.20, 2.73)	2.25 (1.52, 3.32)
Lowest	0.98 (0.79, 1.21)	2.09 (1.65, 2.65)	2.49 (1.99, 3.12)	1.48 (0.96, 2.29)	3.33 (2.45, 4.54)	3.21 (2.54, 4.06)
1997 − 2002						
Highest	1.00	1.03 (0.70, 1.50)	1.41 (0.84, 2.38)	1.00	1.22 (0.67, 2.21)	0.82 (0.12, 5.71)
4	1.14 (0.99, 1.30)	1.22 (0.91, 1.65)	1.27 (0.83, 1.93)	1.34 (1.10, 1.62)	1.47 (0.97, 2.25)	2.11 (0.88, 5.08)
3	1.06 (0.91, 1.25)	1.29 (0.96, 1.71)	1.19 (0.81, 1.75)	1.75 (1.32, 2.32)	1.59 (1.10, 2.30)	1.65 (0.93, 2.93)
2	1.24 (1.02, 1.50)	1.31 (0.97, 1.78)	1.75 (1.29, 2.39)	1.62 (1.07, 2.46)	1.54 (1.07, 2.20)	2.48 (1.80, 3.44)
Lowest	1.36 (1.08, 1.71)	2.14 (1.66, 2.76)	3.65 (3.00, 4.44)	1.47 (1.00, 2.17)	3.34 (2.63, 4.24)	3.87 (3.27, 4.58)
2003 − 2008						
Highest	1.00	1.26 (0.87, 1.81)	1.65 (0.90, 2.99)	1.00	0.84 (0.39, 1.81)	2.27 (0.55, 9.40)
4	1.13 (0.99, 1.28)	1.04 (0.74, 1.46)	1.57 (0.98, 2.51)	1.39 (1.15, 1.68)	0.86 (0.49, 1.52)	3.75 (1.52, 9.28)
3	1.32 (1.15, 1.52)	2.01 (1.56, 2.57)	1.64 (1.08, 2.48)	1.62 (1.24, 2.11)	1.69 (1.15, 2.49)	2.59 (1.36, 4.95)
2	1.43 (1.21, 1.69)	1.67 (1.25, 2.22)	2.09 (1.48, 2.94)	1.71 (1.18, 2.48)	1.65 (1.13, 2.42)	2.25 (1.46, 3.46)
Lowest	1.34 (1.07, 1.67)	2.18 (1.69, 2.82)	4.60 (3.76, 5.63)	1.77 (1.26, 2.47)	3.50 (2.79, 4.40)	3.51 (2.91, 4.24)

Risk ratios and their 95% confidence intervals among 25–59 year-old population in Finland in 1992 − 2008.

Finally, we tested the contrast between the highest and lowest income groups among those living alone and those living with others within each employment status group using models where the time period was adjusted. The results were similar in all employment status groups: those living alone had a larger mortality difference between the extreme income groups compared to those living with others. The risk ratios were 1.34 (1.02-1.75) for those in work, 1.38 (0.89-2.13) for the short-term unemployed and 1.19 (0.44-3.20) for the long-term unemployed. As the confidence intervals indicate the differences were statistically significant only for those in work.

## Discussion

The present study examined the effect on mortality amenable to health-care of three dimensions of social position (economic, social and regional deprivation) that have been suggested by previous literature to be important though their intersections have seldom been explored. The study covered the period 1992–2008, among the working age population aged 25 to 59 years in Finland. We found an inverse income pattern in amenable mortality: the lower the position, the higher the mortality. This result is in line with earlier research both in Finland [15],[26] and elsewhere [16],[19],[23],[24]. Furthermore, being unemployed and living alone increased the mortality risk as has also been reported earlier [15]. Our study adds to the literature by suggesting that a poor labour market situation and living alone increase income group differences. The former result is also in accordance with earlier research on access to health-care, which suggests socioeconomic differences in both access to and content of health-care in relation to need [29]–[31].

For those with a low income and living alone, the effects of unemployment are also likely to be more abrupt and to have a greater impact, as they lack the economic and psychosocial buffers provided by other members of the family. Social networks have been shown to modify and sometimes even prevent some of the effects of unemployment and other negative life events on health; see e.g. [32]. Our findings underline the intersections of different markers of deprivation in showing, on the one hand, how they accumulate and, on the other hand, how they act as a buffer. For example, even prolonged unemployment did not increase the risk of amenable mortality significantly when other buffers (high income and not living alone) were present. The presence of social buffers – such as social support provided by family members and social capital delivered by networks of friends and neighbours – have mostly been suggested to operate by alleviating stress and promoting coping [33]–[35]. Interestingly here the ‘buffering effect’ did not apply to income, as it was found that even among those employed and not living alone, the group with the lowest income had a higher level of amenable mortality. This suggests, firstly, that as a determinant of health income clearly represents a root cause or a cause of causes of ill-health. Secondly, it shows that even among the most privileged who are covered by the well-resourced occupational care, economic deprivation is an influential factor affecting health outcomes. Another potential explanation is reverse causation, i.e. poor health having an impact on income. However, we used family income instead of individual income which is less likely to be affected by ill-health.

One mechanism that may mediate the effects of these deprivation risks is the three tier organisation of ambulatory care in Finland. Having a high income, living in urban or sub-urban areas and employment enable the use of both private and occupational health-care in addition to municipal health-care open to all, whereas for those with a low income, those unemployed and those living in sparsely populated rural areas, municipal health-care is likely to be the only option. Further, during the study period, the resources of municipal health centres remained at the same level, whereas there was a significant increase in the resources for specialist care and private services, including occupational health services. This has led to poorer availability of services and longer waiting times in municipal health centres in many parts of the country. This may in turn lead to delays in diagnosis and poorer continuity of care in chronic conditions, as well as delays in access to specialist care. An earlier study from Finland has reported that visits to the doctor have since the late 1980s consistently been distributed pro-rich when need for services is taken into account [29]. This has been the case in occupational health-care and in private services while in municipal health centres the distribution has been pro-poor and in hospital outpatient services income neutral.

A recent study on all-cause mortality has reported increasing income group differences in life expectancy at age 35 [36]. Further, a study examining amenable mortality in addition to other causes found that in 2006–07 amenable mortality accounted for 9% of differences in life-expectancy between the highest and lowest income deciles among men and 17% among women [37]. Ischaemic heart disease mortality accounted for a fifth of the differences in life expectancy among both genders. While ischaemic heart disease is heavily influenced by health behaviour, it must be borne in mind that part of ischaemic heart disease mortality should also be attributed to health-care.

While the effects of socioeconomic factors on health outcomes result from complex and multilayered processes, this is also true regarding the influence of health-care on health outcomes. The differences found in the current study may arise from differences in access to care, but also in health behaviours increasing the risk of conditions included in the amenable mortality indicator, the incidence or prevalence of these conditions, differences in seeking care and finally in quality of care. Our amenable mortality measure does not take these into account, and we cannot therefore determine whether the differences found are due to poorer access to health-care, less active care seeking, poorer health literacy, differential morbidity or differential quality of care among these socially disadvantaged groups. When interpreting the results, it should also be borne in mind that while the strength of amenable mortality as an indicator is that it relies on a list of conditions in which death could be avoided by timely and effective interventions in health-care, it follows that it sums up death rates from a long list of diseases some of which result in a very small contribution to amenable mortality. In our study, five conditions were the main contributors to amenable mortality: cerebrovascular disease, malignant neoplasms of the breast and of the colon/rectum, diabetes mellitus, and pneumonia. Together they accounted for 70% of amenable deaths in the current study.

A major strength of our study was that the use of individual level register data in the analyses enabled us to avoid ecological bias. The socioeconomic data used in the study form the basis of population censuses while the Finnish Causes of Death Statistics is valid and reliable by international standards [38]. Although some causes of death, such as alcohol poisoning, have been underestimated in routine statistics, the rate of confirmation by autopsy in Finland is high by international comparison (ca 30% for all deaths and about 60% of those of working age). We considered the dimensions of social position that we examined to be factors that could potentially exacerbate the impact of income on amenable mortality. We used living alone as an indirect indicator of social deprivation risk, by which we mean lack of social networks. Clearly this is a crude proxy that does not take into account people’s social networks outside their home or the content or adequacy of the social support provided by the family. Unfortunately, the register data used in this study did not contain more detailed information about this dimension of social position. Living in sparsely populated rural areas was considered to increase the risk of deprivation, given the evidence on accumulation of disadvantages from poverty and unemployment in these types of rural areas both internationally [9] and in Finland [10]. One challenge in the analyses was to deal with several measures of social position that are likely to be correlated. Although we stratified the data by our indicator of social deprivation to minimise problems caused by this, an expected association between, for instance, income and unemployment is obvious in the results. A limitation of the study was that we were not able to follow the employment status of people across years in our data, and cannot therefore determine the share of long-term unemployed who are permanently (across years) unemployed with reduced income. We have taken these limitations into account when interpreting the results.

Recent studies on amenable mortality have criticised the use of amenable mortality especially in international comparisons as an indicator for the performance of health-care [39],[40]. These studies were not able to identify strong associations between changes in mortality to specific conditions included in amenable mortality and the introduction of specific health-care innovations [39] or between inequalities in mortality and access or quality of health-care [40]. Nevertheless, our results suggest that amenable mortality as a broad category may have value at least in within-country analyses in identifying those clusters of social and economic disadvantage that may be associated with increased risk of mortality in treatable conditions. An earlier study suggested that 60 − 80% of amenable deaths are deaths that could be avoided by action in primary care [26]. These rely on early detection and treatment of conditions that lead to poor health outcomes, such as type 2 diabetes. This suggests that there is room for primary care to develop methods for prevention, early detection and treatment of these conditions to reduce the mortality risk in groups experiencing multiple risk of social and economic disadvantage.

## Conclusions

Our results suggest that in order to prevent and treat early phase conditions that otherwise may lead to premature and unnecessary deaths, especially municipal health centres need to reach and deliver high quality care to people of different social backgrounds. Our results also call for joint action within both health-care and social services, since health services cannot alone deal with the risks that arise from accumulated social disadvantage.

## Competing interest

The authors declare that they have no competing interest.

## Authors’ contributions

KM contributed to the conception and design of the study, planning of analyses and drafted the manuscript. MA contributed to the conception and design of the study, performed the statistical analyses and took part in the revision of the manuscript for important intellectual content. RS contributed to the conception and design of the study, planning of analyses, drafting the manuscript and took part in the revision of the manuscript for important intellectual content. SK contributed to the conception and design of the study, planning of analyses and took part in the revision of the manuscript for important intellectual content. All authors read and approved the final manuscript.

## Authors’ information

KM holds a PhD in sociology and works as a research director in the National Institute for Health and Welfare (THL). A main focus of her research has been equity issues in health services research. MA holds a MSc in biostatistics and works as a statistician in THL. He has long experience in analysing register based data. RS holds a PhD in statistics and works as a research director at THL. A main focus of his research has been developing methodology for register based health services research. SK holds a PhD in sociology and works as a director of department at THL. A main focus of his research has been regional welfare differences.

## Additional file

## Supplementary Material

Additional file 1: **List of causes of death considered amenable to health-care and the corresponding ICD-9 and ICD-10 codes.** The file contains all the causes of death considered amenable to health-care. Click here for file
