# Supplementary material for: Multiple social disadvantage does it have an effect on amenable mortality: a brief report
Source: Int J Equity Health. 2014 Aug 1;13:67. doi: 10.1186/s12939-014-0067-5 (PMC4237818; doi:10.1186/s12939-014-0067-5)
Supplement: Additional file 1: — List of causes of death considered amenable to health-care and the corresponding ICD-9 and ICD-10 codes. The file contains all the causes of death considered amenable to health-care. [file s12939-014-0067-5-S1.pdf]

Additional file 1. List of causes of death considered amenable to health care and corresponding ICD codes.

Cause of death	Age[1]	ICD-9	ICD-10
Diphtheria, Tetanus, Poliomyelitis, and Varicella	1–74	032, 037, 045, 052	A35–36, A80, B01
Rubella	1–74	056	B06
Scarlatina	1–74	034	A38
Meningococcus	1–74	036	A39
Erysipelas	1–74	035	A46
Legionellosis	1–74	482.84	A48.1
Malaria	1–74	084	B50–54
Streptococcal pharyngitis	1–74	034.0	J02.0
Cellulitis	1–74	681–682	L03
Tuberculosis	1–74	010–018, 137	A15–A19, B90
Melanoma of skin	1–74	172	C43
Malignant neoplasm of skin	1–74	173	C44
Malignant neoplasm of breast	1–74	174	C50
Malignant neoplasm of cervix uteri	1–74	180	C53
Malignant neoplasm of cervix uteri and body of uterus	1–44	179, 182	C54–55
Malignant neoplasms of colon and rectum	1-74	153-154	C18-C21
Malignant neoplasm of bladder	1–74	188	C67
Benign tumours	1–74	210–229	D10–36
Hypertensive disease	1–74	401–405	I10–13, I15
Cerebrovascular disease	1–74	430–438	I60–69

Diseases of the thyroid	1–74	240–246	E00–07
Diabetes mellitus	1–49	250	E10–14
Epilepsy	1–74	345	G40–41
Asthma	15–49	493	J45–46
COPD	15–49	490–492, 496	J40–44
Septicaemia	1–74	038	A40–41
Malignant neoplasm of testis	1–74	186	C62
Hodgkin's disease	1–74	201	C81
Leukaemia	1–44	204–208	C91–95
Rheumatic and other valvular heart disease	1–74	390–398	I01–09
Influenza	1–74	487	J09–11
Pneumonia	1–74	480–486	J12–18
Peptic ulcer	1–74	531–534	K25–28
Appendicitis	1–74	540–543	K35–38
Abdominal hernia	1–74	550–553	K40–46
Cholelithiasis and cholecystitis	1–74	574–575.1	K80–81
Nephritis, nephrosis, and nephropathy	1–74	580–589	N00–N09, N17–N19, N25–N27
Obstructive uropathy and prostatic hyperplasia	1–74	592, 593.7, 594, 598.0–598.1, 598.8-598.9, 599.6, 600	N13, N20–N21, N35, N40
Maternal death	All	630–676	O00–O99
Congenital cardiovascular anomalies	1–74	745–747	Q20–Q28

[1] In our study, deaths of people under 25 years and over 59 were excluded from the analyses.
